# Rapid Environmental Impact Assessment of Penicillin G in a Veterinary Product Using an Original Software Method and Monitoring by SPE-Online-UHPLC-MS/MS

**DOI:** 10.3390/molecules28176227

**Published:** 2023-08-24

**Authors:** Viviana Carmen Ciucă, Victor Viorel Safta, Carmen Otilia Rusănescu, Gigel Paraschiv, György Deák, Mihaela Ilie, Sorin Cănănău

**Affiliations:** 1 Faculty of Biotechnical Systems Engineering, University of Science and Technology Politehnica Bucharest, 313 Spl. Independentei, 060042 Bucharest, Romania; 2 National Institute for Research and Development in Environmental Protection, 294 Spl. Independentei, District 6, 060031 Bucharest, Romania; 3 Faculty of Mechanical and Mechatronic Engineering, University of Science and Technology Politehnica Bucharest, 313 Spl. Independentei, 060042 Bucharest, Romania

**Keywords:** Penicillin G, risk assessment, software method, SPE-online-UHPLC-MS/MS

## Abstract

Veterinary antibiotics have become a major concern due to potential environmental effects. This study presents an investigation of the exposure and environmental effects of the veterinary medicinal product in powder form, administered in the drinking water of piglets, chickens and turkeys, containing 250 mg/g penicillin G (benzylpenicillin potassium), performed according to the European Medicines Agency (EMEA) guideline and the results obtained by an analytical method based on online solid-phase extraction, ultra-high performance liquid chromatography coupled to a triple quadrupole mass spectrometer (SPE-online-UHPLC-MS/MS). The study presents the determination of the environmental risk and through an original, interactive, fast software method, created on the basis of a proprietary calculation algorithm that goes through all the prescriptions and recommendations of the EMEA guide. The results demonstrated that the concentration value for penicillin G determined in surface water by SPE-online-UHPLC-MS/MS is much lower than that predicted by calculation (predictable concentration in surface water, PECsurface water = 37.66 µg/L and the concentration SPE-online-UHPLC-MS/MS = 0.032 µg/L). Both results lead to a sub-unit risk quotient (R) indicating that the treatment carried out with the considered veterinary product does not present any risk to the environment.

## 1. Introduction

Pharmaceutical pollution of the environment has become a major concern due to potential effects on human and animal health. Recent worldwide research on the impact of veterinary drugs on environmental organisms and human health has revealed low levels of veterinary drugs in soil, surface water and groundwater [1,2,3,4,5].

In environmental assessments of veterinary pharmaceuticals, required by the U.S. Food and Drug Administration (FDA) since 1980 and in the European Union since 1997, the effects of veterinary drugs on biota are analyzed [6,7]. The results of the studies carried out in these evaluations are accessible in numerous publications and provide a wealth of information on the environmental fate and effects of veterinary medicinal products [8,9]. In this study, we use available data to address major questions and concerns regarding the environmental impact of penicillin G following administration of a veterinary pharmaceutical product. To understand the potential ecological risk of penicillin G and to develop management strategies to reduce it in soil, water and sediment matrices, one of the most important steps is the determination of environmental concentrations and the assessment of their effects on biota [10]. The release of antibiotics into the environment, especially in natural water systems, contributes to the development and global spread of antibiotic resistance. Human health risk assessment of environmental antibiotic residues and antimicrobial resistance is a growing public health concern. In this environmental impact study, we chose potassium benzylpenicillin because it is considered the leading penicillin and still retains its importance in the treatment of infections with susceptible germs. At this time there are approved conditioners on the market, for example: Aviapen^®^ 250 mg/g (Serumwerk Bernburg A.G.), PenAqua Sol-G^®^ (Bimeda); Pot-Pen^®^ (Vetoquinol) and Penicillin G potassium USP soluble powder (BioAgriMix). Penicillin G has a much higher absorption rate in the intestine (60%), which favors a quick and decisive action on *Clostridium perfringens*.

Thus, in a study carried out in 2008, Gadbois and colleagues observed the role of penicillin G potassium salt (from the Pot-Pen product) administered in an extralabel manner, i.e., orally in drinking water, in doses of 0.2 (297,000 IU) and 0.4 (594,000 IU) g/L of water, in the treatment of clinical and subclinical conditions induced in vitro by *Clostridium perfringens* in broilers. The results validated the increased efficiency of both doses administered in necrotic enteritis produced by *C. perfringens* and finally, the decrease in the mortality rate. As a protective measure, benzylpenicillin is not used in birds from which eggs intended for human consumption are obtained.

The Merck Veterinary Manual, in the Birds chapter, states that in the treatment of necrotic enteritis, the most common, drug administrations are done orally in the drinking water with the drugs: bacitracin between 5 and 7 days, penicillin 5 days and lincomycin 7 days. In these cases, medicated water will be the only source of water.

The ANTIBIOTOX project funded by the French ANR (Agence National de Recherche, followed during the period 2018–2022) studied the biodegradation pathways and the estimation of the ecotoxicological impact in the aquatic and terrestrial environment of antibiotics. Spain was chosen as an example for the use of veterinary medicines (European Union’s Horizon 2020 Research and Innovation programme, Grant/Award).

As major antibiotics, tetracyclines and beta-lactams contributed more to soil vulnerability than less commonly used antibiotics.

In studies conducted around the world on different food products of animal origin, penicillin residual concentrations below 10 µg/kg were detected in meat (Vietnam 2015) and 353 µg/kg in milk (Nigeria 2018) [11,12,13]. Research on the occurrence of pharmaceuticals in the environment has been carried out through chemical analyzes and risk assessment studies [14,15]. Environmental risk assessment is usually based on toxicity tests performed on established standard organisms such as fish, algae or other suitable sensitive species. The results of these configurations provide information on the toxicity of substances to target and non-target organisms [16]. Antibiotics are not regulated by current European water quality standards, which require evidence of their widespread contamination of the environment [17]. An environmental risk assessment study of penicillin drugs administered to animals considers the possibility that such uses may increase the incidence of penicillin-resistant *Enterococcus faecium* in human infections. This study suggests that the current use of penicillin in livestock in the United States poses very low (possibly zero) risks to human health [18].

The first antimicrobial compound, penicillin, was discovered by Alexander Fleming in 1928, when he observed that the fungus *Penicillium notatum* destroyed colonies of *Staphylococcus aureus*. In 1939, Howard Florey, professor of pathology at Oxford University, starting from Fleming’s article, succeeded in purifying penicillin and doing tests on animals. Penicillin is based on a heterocyclic system formed by the condensation of an azetidine-2-ionic ring with a thiazolidine ring, having three carbon atoms from the asymmetric positions 2, 5 and 6, which is why penicillin has optical activity [19].

Once the pharmaceutical product containing penicillin G is used and subsequently absorbed by animals, the active substance is metabolized. The degree of metabolism depends not only on the type of substance but also on the treated species, as well as on its age and health. The unmetabolized fraction is eliminated in feces and urine, reaching the environment (mainly soil and water) [20]. A simple and sensitive method for the rapid separation and detection of penicillin G in water samples is high-performance thin-layer chromatography (HPTLC), an ideal technique for the determination of some antibiotics because it is an inexpensive, rapid, easy-to-use technique that does not involve complicated maintenance and offers the possibility of simultaneous analysis of several samples. In a study on the behavior of penicillin G in wastewater treatment processes and in the aquatic environment, an analytical method was developed for the simultaneous detection of penicillin G and five degradation products using liquid chromatography-electrospray ionization mass spectrometry (LC-ESI/MS). The final penicillin G concentrations in the treated water were 1.68 ± 0.48 µg/L. In the receiving river, the concentration of penicillin G decreased from 0.31 ± 0.04 µg/L. The main degradation products of penicillin G in surface waters were found to be penyloic acid, penicilloic acid and isopenylic acid, which occupied 65.8%, 20.4% and 12.9% of the total concentration, respectively [21,22].

The study we present aims to understand the biotic and abiotic processes underlying the release, environmental fate and effects of penicillin G from an oral powder veterinary pharmaceutical product administered in the drinking water of piglets, chickens and turkeys. The complex investigation of penicillin G, going through the European guideline EMEA/CVMP/ERA/418282/2005-Rev1, as well as the analytical method liquid chromatography-tandem mass spectrometry, allows an assessment of the environmental risks it presents [23,24]. Moreover, in the paper we present an original, interactive, fast software method, created on the basis of a proprietary calculation algorithm that goes through all the prescriptions and recommendations of the EMEA guide, determines the predictable concentrations in soil, water and sediment for all treated animal species and finally, environmental risk. The created method reduces the time of environmental impact assessment.

## 2. Results

The results of the analysis of the impact of penicillin G on the environment are presented by initially going through all the steps and calculations provided in the ECHA guide, then by applying the original software method. The monitoring of penicillin G in surface waters by SPE-Online-UHPLC-MS/MS allows a comparative analysis between predicted and actual results.

### 2.1. Environmental Risk Assessment in Accordance with the EMEA/CVMP/ERA/418282/2005-Rev1 Guide

#### 2.1.1. Calculation of PEC (Exposure Analysis)

The exposure assessment was carried out using the total residue approach. The total amount of the applied dose, excreted by the treated animal, without the metabolism data, was taken into account. In phase I, the predictable concentration of penicillin G in the soil (PECsol initial) was calculated, after the administration of the analyzed product to the species and categories of intensively raised animals taking into account the maximum limit of the specific amount of nitrogen that is administered on the soil in the EU, lrN = 170 kg N/ha, the gross density of the dry soil, ρbsu = 1500 kg/mc, and the depth of penetration into the soil of the active ingredient from the analyzed product, aps = 0.05 m. The results obtained for each species were:

- PECsol initial chicken = 354.78 µg/kg;
- PECsol initial turkey = 176.8 µg/kg;
- PECsol initialf piglet = 868.88 µg/kg

Since the PECsol initial values for each target species are greater than 100 µg/kg, the environmental risk assessment continues in phase II. The most unfavorable value of the PEC soil initial will be used, namely the one for piglets.

#### 2.1.2. Calculation of PECwater, PECsediment

The predictable concentrations of penicillin G in ground water (PECground water), surface water (PECsurfacewater) and in sediment (PECsediment) were calculated, taking into account the predictable concentration in the soil at a depth of 20 cm of the active ingredient (PECsoli20cm), molar mass (MW = 372.48), solubility in water (SOL = 307 mg/L), vapor pressure (VP = 2370) and distribution coefficient water–organic carbon (Koc = 421l/kg) (Table 1) [24,25,26,27].

For the calculation of the predictable concentrations of penicillin G in soil, water and sediment, the pig species was taken into account, with the most unfavorable value of the initial predictable concentration in the soil.

#### 2.1.3. Calculation of PNEC (Effect Analysis)

The predicted no-effect concentration (PNEC), the concentration of penicillin G that marks the limit below which adverse effects of exposure in an ecosystem are not measured, was calculated depending on the type of data used; the rating factor was used to account for the confidence of toxicity data being extrapolated to an entire ecosystem. The result of experimentally determined effects was divided by an appropriate assessment factor (AF). Depending on the type of data used, the rating factor was used to account for the confidence of toxicity data being extrapolated to an entire ecosystem. Some organisms, such as blue-green algae, have been found to be highly sensitive to penicillin, exhibiting a NOEC (no-observed-effect concentration) as low as 0.78 mg/L. Other organisms, such as the zooplankton, D. magna and the green alga S. capricornutum, showed NOECs at concentrations as high as 300 mg/L, suggesting that they were quite insensitive to penicillin. Data used in the PNEC calculation are NOEC (Algae growth inhibition): 10 mg/L, EC50–Median Effective Concentration (activated sludge inhibition test): 100 mg/L, and NOEC earthworm: 2000 mg/kg (Table 2) [28,29,30,31].

In the case above, the calculated predicted no-effect concentration in water (PNECa FA) is 1 mg/L. By the evaluation factor method, the calculated predicted no-effect concentration in soil (PNECsoilFA) is 20 mg/kg and the calculated predicted no-effect concentration in sediment (PNECsedimentFA) is 1 mg/kg [31,32,33,34,35].

PNECsoil = (0.1176 + 0.01764 × Koc) × PNECwater = 7.69 mg/kg(1)

PNECsed = (0.783 + 0.0217 × Koc) × PNECapa = 9.93 mg/kg(2)

The predicted no-effect concentration in soil calculated by the partition equilibrium method (PNECsoilEP) is 7.69 mg/L (1) [30]. The predicted no-effect concentration in sediment calculated by the partition equilibrium method (PNECsediment EP) is 9.9 mg/L (2) [30]. The lowest value for soil PNEC and sediment PNEC will be used in the following calculations.

#### 2.1.4. Calculation of Rsoil, Ground Water, Surface Water and Sediment (Risk Analysis)

PECs initially calculated based on the total residue approach are compared with the PNEC derived from the set of toxicity tests (Table 3).

The values obtained for PEC and PNEC in soil, water and sediment lead to a risk R ˂ 1, which indicates that the risk to the environment when administering the analyzed product is acceptable and the evaluation stops here.

### 2.2. Environmental Risk Assessment of Penicillin G Using the Software Method

After running the interactive software, created according to its own algorithm, in accordance with the EMEA/CVMP/ERA/418282/2005-Rev1 [23] guideline, after entering the data on the administration of the veterinary product, the physicochemical characteristics and the ecotoxicological data of penicillin G, the following results were obtained:

- The matrices of the predictable concentration in the soil (PECsoilinitial), the predictable concentration in groundwater (PECgroundwater), the predictable concentration in surface waters (PECsurfacewater) and the predictable concentration in the sediment (PECsediment) contain the calculated results for all species and categories of intensively raised animals (pigs, broilers and turkeys) and treated with the analyzed product, according to the calculation formulas provided in the guide [23].

**Figure 1:**
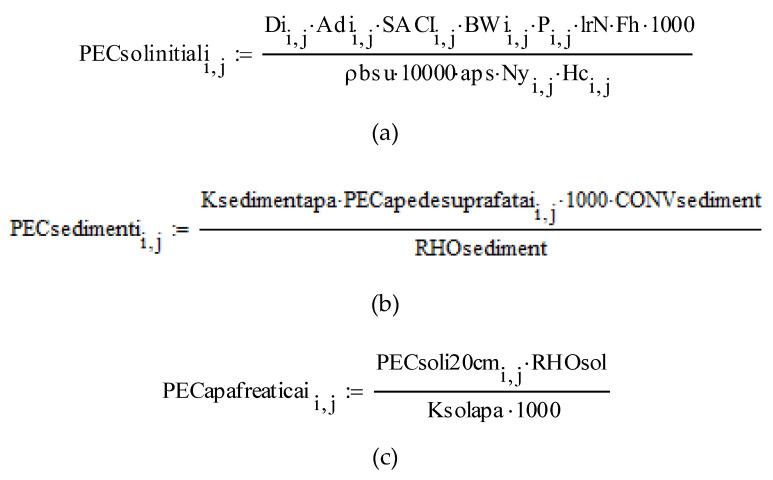

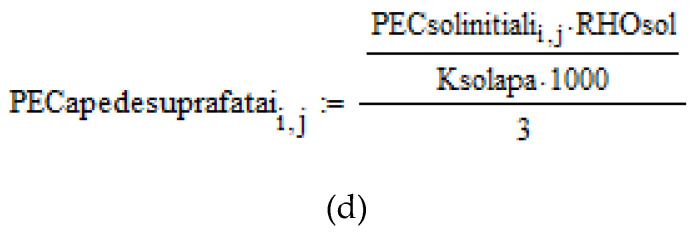
The matrix relations (**a**–**d**) images taken directly from the software.

**Figure 2:**
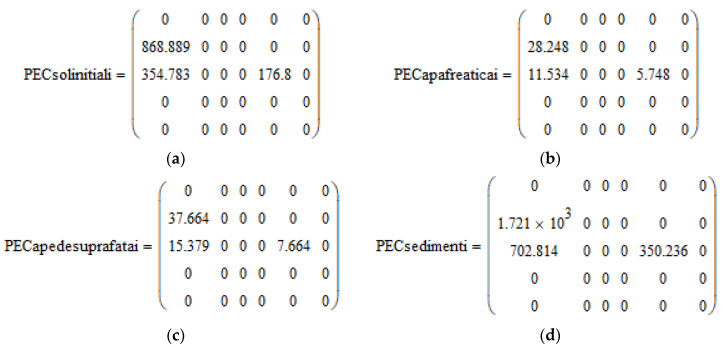
Matrix PEC calculation—*software aspects. (***a**) Matrix PECsoilinitial, [μg/kg]; (**b**) matrix PECgroundwater, [μg/L]; (**c**) matrix PEC surface water, [μg/L]; and (**d**) matrix PEC sediment, [μg/kg]—images taken directly from the software.

**Figure 3:**
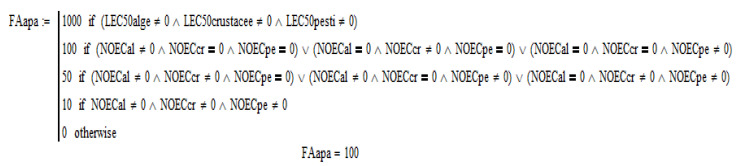
FA evaluation factor for PNEC water–software aspect.

**Figure 4:**
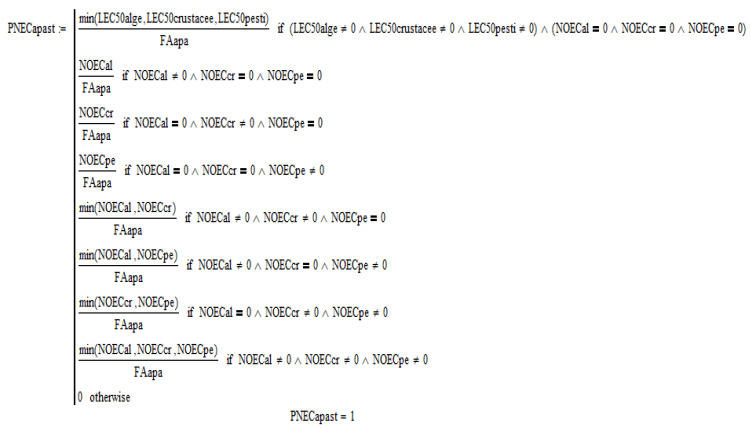
PNECwater concentration [mg/L]–software aspect.

**Figure 5:**
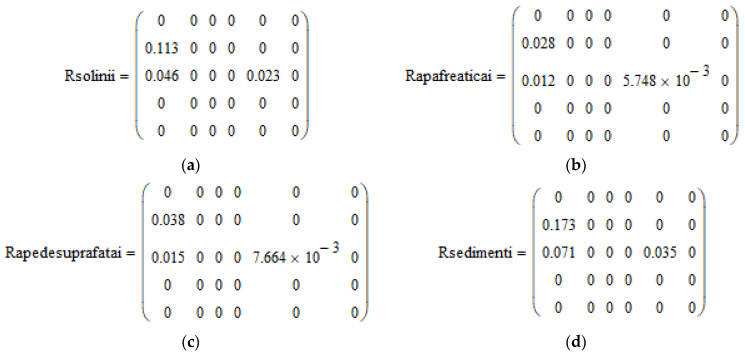
Matrix R calculation–*software aspects.* (**a**) Matrix Rsoil; (**b**) matrix Rgroundwater; (**c**) matrix Rsurface water; and (**d**) matrix Rsediment.

Images from the software are presented in the Figure 1, Figure 2, Figure 3, Figure 4 and Figure 5.

- After entering all available significant values of penicillin G toxicity and the conditions for the evaluation factor method, *FAwater* = 100, *PNECwater =* 1 mg/L (Figure 3 and Figure 4), *FAsoil* = 100, *PNECsoil =* 20 mg/kg were obtained; and by the partition balance method *PNEC soil =* 7.69 mg/kg, *PNEC sediment =* 9.93 mg/L [30].

- The assessment of the initial risks of penicillin G after the administration of the analyzed product, quantified by the indicators of Rsoil, Rgroundwater, Rsurfacewater, and Rsediment, are calculated for all species and categories of intensively raised animals according to the calculation formulas provided in the guide (7), (8), (9) and (10), *software aspects* [23,36,37,38,39,40], (Figure 5a–d).

### 2.3. Determination of Penicillin G Content in Surface Waters by SPE-Online-UHPLC-MS/MS

**Figure 6:**
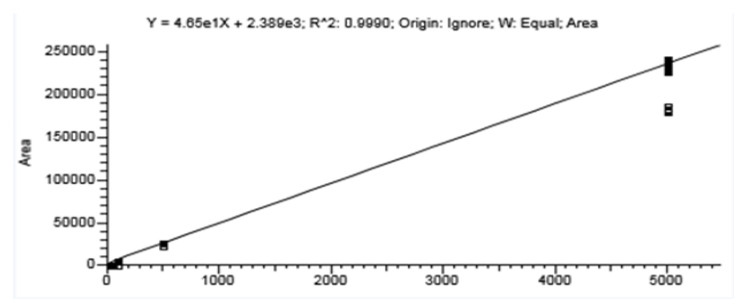
Penicillin G calibration curve.

**Figure 7:**
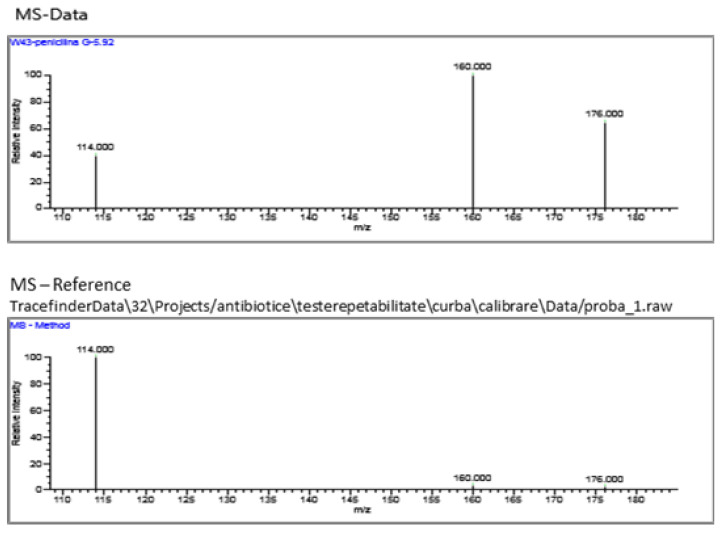
Calibration curve—repetability tests.

The analytical method used in this paper was evaluated in terms of linearity, repeatability, accuracy and sensitivity. The standard solution of 100 μg/mL penicillin G purchased from the manufacturer was used to prepare a stock solution of 1 μg/mL in 1:1 (*v*:*v*) water to acetonitrile. A solution of 0.01 μg/mL (10 ng/mL) was prepared from the stock solution, which was further diluted to 5 corresponding levels of 4 pg/mL, 20 pg/mL, 100 pg/mL, 500 pg/mL and 5000 pg/mL (4 ng/L, 20 ng/L, 100 ng/L, 500 ng/L and 5000 ng/L). The standard calibration curve was drawn based on the results obtained with the standard calibration solutions. The linearity of the response was verified. Penicillin G concentration was calculated using the standard calibration curve. The calibration curve obtained for penicillin G was linear, with a correlation coefficient R^2^ > 0.999 (Figure 6). The relative standard deviation (RSD) was 2.5% RSD for repeatability and 3.8% RSD for reproducibility. The precision limit <10% RSD was met, indicating good precision of the method. The method validation was performed by analyzing 10 replicates at five different concentrations (4 ng, 20 ng, 100 ng, 500 ng and 5000 ng). A mixed standard working solution with antibiotic concentrations ranging from 4 to 5000 ng/L was set up for detection and analysis. The limit of detection and the limit of quantification were calculated by the method based on the calibration curve. The residual standard deviation of the areas, as standard deviation (σ), was calculated. The limit of detection was calculated with the formula LOD = 3.3 σ/p, where p is the slope of the curve and the limit of quantification LOQ = 10 σ/p (σ = 0.43; p = 46.5). The limit of detection (LOD) for penicillin G tested in the water sample was 0.03 ng/L and the limit of quantification (LOQ) 0.09 ng/L. The on-line SPE system eliminates human SPE errors and provides very good reproducibility of the method (Figure 7).

Analysis report data:injected concentration: 32.452, retention time: 5.92, area: 1748, and height: 2032.


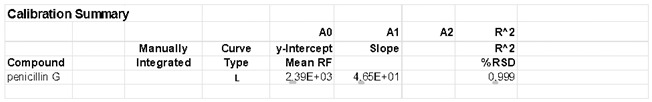


TraceFinder 3.2 software, processes the data both for the realization of the calibration curves, the validation of the method and the processing of the acquisition data for the identification of the pharmaceutical compounds from different samples and respects the requirements of the European guidelines for the validation of the methods.

**Figure 8:**
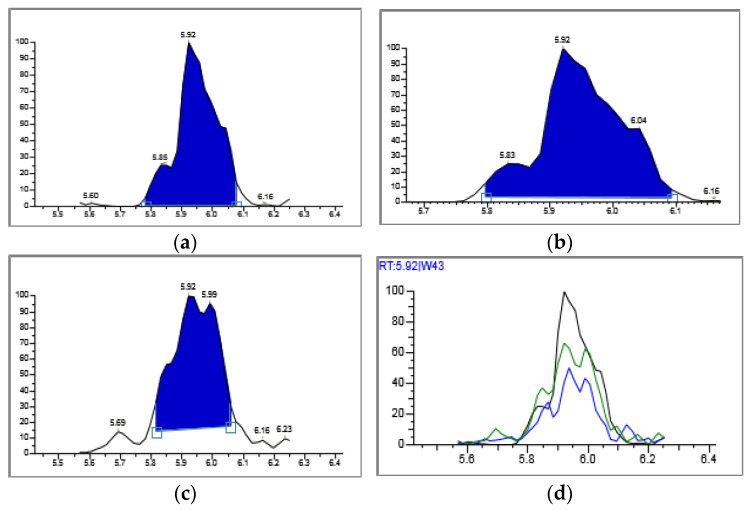
Thermo Scientific Instrument analysis report. Confirmatory ion chromatograms for Penicillin G: (**a**) Quan Peak: 335.200- > 114.000 mz. (**b**) Qual Ion 1: 335.200- > 160.000 mz. (**c**) Qual Ion 2: 335.200- > 176.000 mz. (**d**) Overlay-chromatograms of the 3 confirmatory ions for penicillin G.

The results of the optimized ionization mode, fragmentation voltages, collision energies and chromatographic retention time for penicillin G are given in the Thermo Scientific Instrument analysis report (Figure 8).

**Figure 9:**
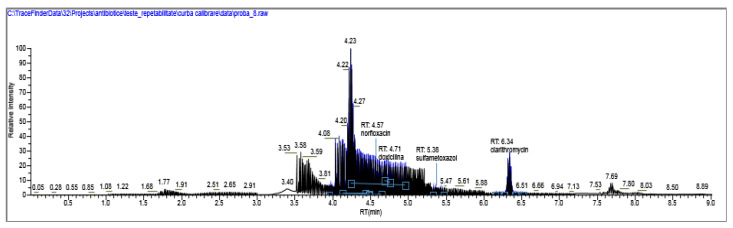
Identification, dosage of pharmaceutical pollutants from the Ialomița river—sample 1.

**Figure 10:**
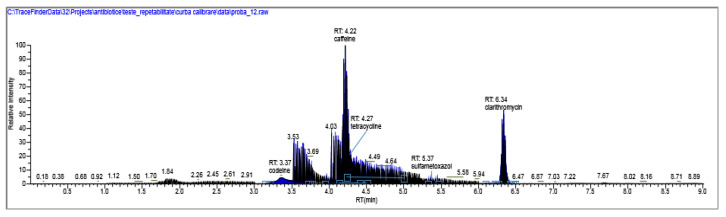
Identification, dosage of pharmaceutical pollutants from the Ialomița river—sample 2.

**Figure 11:**
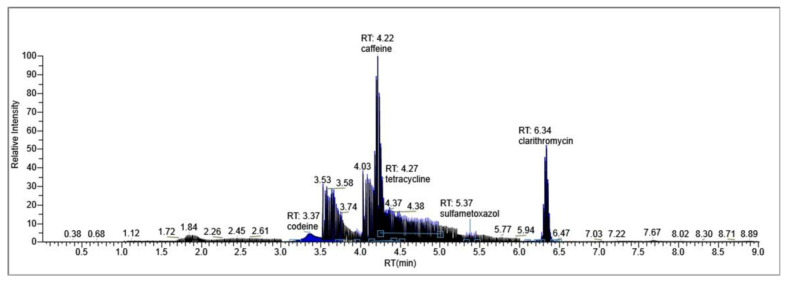
Identification, dosage of pharmaceutical pollutants from the Ialomița river—sample 3.

**Figure 12:**
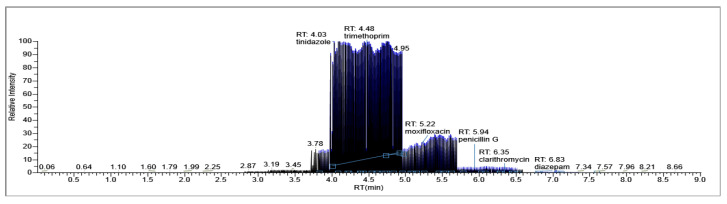
Identification, dosage of pharmaceutical pollutants from the Ialomița river—sample 4.

The results of penicillin G monitoring from the analyzed surface water samples are presented in Figure 9, Figure 10, Figure 11 and Figure 12.

Penicillin G was not detected in the surface water samples analyzed, 1 and 2, taken on 15 and 16 March 2023 (Figure 8 and Figure 9).

Penicillin G was identified and dosed in a concentration of 0.032 µg/L (32 ng/L), only in sample 3, taken on 17 March 2023 (Figure 10).

In sample 4 of surface water, taken on 18 March 2023, penicillin G was not detected. (Figure 12).

Figure 9, Figure 10, Figure 11 and Figure 12 show the results corresponding to the identification of each compound according to RT, the confirmation of each compound according to MS for Precursor Ion and Product Ion is presented in the confirmation report for each analyzed sample (Figure 8).

## 3. Discussion

The latest data reported by the European Food Safety Authority (EFSA) shows that residues of veterinary drugs and other substances found in animals and food of animal origin continue to decrease in the European Union and compliance levels are increasing. In 2021, the percentage of non-compliant samples was 0.17%, the lowest figure recorded in the last 12 years when non-compliance varied between 0.19% and 0.37%. The figure for 2020 was 0.19%. The report (EFSA) includes hormones, antibacterials, environmental contaminants, banned substances and other veterinary drugs.

Analyzing the impact of pharmaceutical substances on the environment, the European Union Commission meeting in Brussels (September 2020) considered effective measures to reduce the impact of pharmaceutical substances on the environment and additional research to regulate the level of pharmaceutical residues in water legislation to be necessary. The assessment of the environmental risks of veterinary medicinal products in groundwater according to the current provisions of the European Union suggests an approach based on the comparison between the calculated concentration in ground water (PEWater table) and an arbitrarily established threshold concentration of 0.1 μg/L, which represents the upper limit of pesticide concentration in groundwater in the EU. If the calculated PEHeadwater does not exceed the threshold, then the risk is considered acceptable. The concentration of 0.1 μg/L is assumed to be safe by default for both humans and exposed organisms in groundwater.

β-lactam antibiotics (penicillin, the first β-lactam antibiotic discovered) are not usually detected in the aquatic environments analyzed in Europe due to their high lability to heat, light, extreme pH, metal ions, oxidizing and reducing agents, nucleophiles and solvents, such as water, which lead to their hydrolysis under ambient temperature and pH conditions. However, based on several studies carried out in the EU, the ranges of β-lactam concentrations in water were determined, namely: 18–6196 ng/L in the influent of used water treatment plants, 47–1205 ng/L in the eluent of wastewater treatment plants and 3.57–552 ng/L in surface waters [17,41,42,43,44,45,46].

Based on different application objectives (human or veterinary medicine), antibiotics, classified as priority and very important categories by the World Health Organization (WHO) and widely used in Romania, penicillin G was selected as the target compound in the present study. Antibiotic contamination can lead not only to ecotoxicity but also to antibiotic resistance in the aquatic environment.

This work focuses on understanding the biotic and abiotic processes that are the basis of the environmental impact of penicillin G from a veterinary drug. Such studies are very important to detect penicillin G residues and the potential indirect effects of environmental exposure on ecological and human health.

Penicillin G was not detected in the investigated surface water samples. Traces of penicillin G were only detected in one sample. Through this study, SPE-online-UHPLC-MS/MS proved to be a powerful analytical tool that allows highly sensitive detection of antibiotics in surface waters, even when they are present in trace amounts (ng/L). This type of analysis will provide important insights into the occurrence and distribution of antibiotics (and other pharmaceuticals) in drinking water supplies and could help to impose appropriate limits on the amounts of these chemicals that are allowed to enter the environment. The method developed for the quantification of penicillin G was also used for the detection of other pharmaceutical pollutants, namely 50 pharmaceutical compounds from the category of antibiotics, estrogen hormones, anti-inflammatories, analgesics, beta blockers, benzodiazepines, antidepressants and antiepileptics.

In the environmental risk assessment of penicillin G according to the EMEA guideline [22], the predicted concentration of penicillin G in surface water (37.66 µg/L) is higher than the experimentally determined one (0.032 µg/L), but the risk of the calculated environment (R) is a subunit and indicates that the treatment carried out with the considered veterinary medicinal product does not pose any risk to the environment.

The software method created for the purpose of assessing the environmental risk of penicillin G follows and concretely explains the steps of the calculation algorithm, having incorporated all the data from the specific guide, and it is no longer necessary to consult it during the analysis. The software is very easy and convenient to operate, allowing easy entry of all data regarding the analyzed veterinary medicinal product and allows complex environmental risk assessment analyzes to be performed in short periods of time.

The environmental impact assessment methods presented in this paper may form the basis of future assessments for other classes of active substances in veterinary pharmaceutical products.

## 4. Materials and Methods

### 4.1. Environmental Risk Assessment of Penicillin G According to Guideline [22]

The environmental risk assessment goes through the following stages:

1. The scheme of the decision tree regarding penicillin G, the active substance of the considered veterinary product, which contains 250 mg/g penicillin G and is administered as a powder in the drinking water of piglets (25 mg/kg body weight, for 3–4 consecutive days), chickens and turkeys (10 mg/kg body weight, for 3–4 consecutive days.
2. The initial PECsol calculation for each target species and technological category. If the initial PECsol values for each target species are greater than 100 µg/kg, the environmental risk assessment carried out according to phase I, must continue to phase II. The most unfavorable PECsol value will be used in the following determinations.
3. Based on the acquired data regarding the physico-chemical properties, the evolution and behavior in soil, water and sediment adsorption in the soil, PECground water, PEC surface water, and PECsediment are calculated.
4. Based on the acquired toxicity data, PNECsol, PNECwater and PNECsediment are calculated, using the factor evaluation method and the partition balance method [30].
5. For the initial risk analysis R, the PECs initially calculated based on the total residue approach are compared with the PNECs derived from the set of toxicity tests.
6. If R < 1, the assessment ends, considering that there would be no risk to the ecosystem.
7. If R > 1, refinements are made of PECs based on metabolism, excretion and degradation in feces and degradation in soil leading to the establishment of the refined risk.
8. If the refined risk, Rraf > 1, proceed to step B of phase II and refine the PNECs.

### 4.2. Environmental Risk Assessment of Penicillin G Using the Created Software Method

The penicillin G environmental risk assessment software is in accordance with the prescriptions and recommendations of the EMEA guide, it is developed on the basis of its own calculation algorithm, and all the specific data from the guide are included and explained, it is no longer necessary to consult it during risk analysis [22,36,37]. The software method only involves entering the product administration data, the physico-chemical and ecotoxicity data of penicillin G and reading the displayed results for all indicators calculated according to the EMEA guide. It should be mentioned that the software allows the determination of PNECsol and PNECsediment by two methods, the evaluation factor method and the partition balance method, with the value taken in the calculations being the minimum value resulting from the application of the two methods. The software displays individual, cumulative and maximum values of all calculated indicators.

### 4.3. Determination of Penicillin G Content in Surface Waters by SPE-Online-UHPLC-MS/MS

#### 4.3.1. Sampling and Preparation of Water Samples

The identification and dosing of penicillin G was carried out from the Ialomița river, the Buzău-Ialomița hydrographic basin, in the area downstream from the city of Slobozia. Livestock farms and agricultural land are located upstream of the sampling area, as well as the sewage treatment plant in the city of Slobozia. The collection of the 4 samples was carried out daily between 15 and 18 March 2023. The water sample was taken from approximately 0.5 m below the water surface and stored in a brown bottle for analysis. After collection, the sample was stored in the ice box and delivered on the day of sample collection to the laboratory, where it was stored at 4 °C in the laboratory refrigerator until analysis. The surface water sample was filtered through 0.2 µm polyethersulfone (PES) filters (Sartorius Stedim Biotech GmbH, Göttingen, Germany) before direct injection into the SPE-online-UHPLC-MS/MS system.

#### 4.3.2. Equipment

Identification of antibiotics was performed using liquid chromatography coupled with tandem mass spectrometer SPE-online-UHPLC-MS/MS Thermo Fisher Scientific™ EQuan MAX Plus™UltiMate 3000 system connected to a TSQ Quantiva triple quadrupole mass spectrometer equipped with an electrospray ionization source in positive mode and TraceFinder 3.2 software (US) for data acquisition and processing.

The technique used is the most sensitive and selective compared to other frequently used analytical techniques, allowing for the detection and quantification of emerging pollutants in the category of pharmaceuticals at ng/L level.

#### 4.3.3. Reagents

The reference standards used for this analysis were penicillin G potassium salt (≥97% purity), produced by Dr. Ehrenstorfer, Germany. Ultra-pure water was provided by VWR Chemicals and used in preparing all standard solutions. HPLC grades acetonitrile and formic acid for LC/MS were provided by Scharlau Chemie SA and VWR Chemicals.

#### 4.3.4. Metoda SPE-Online-UHPLC-MS/MS

Method developed in this study for the identification and dosing of penicillin G by SPE-online-UHPLC-MS/MS method meets the requirements at the EU level regarding the detection limit. Identification and quantification were performed under selected reaction monitoring (SRM) mode, recording the transitions between the precursor ion and the two most abundant product ions for each target analyte, thus achieving three identification points per compound according to Commission Decision 2002/657/CE regarding the performance of analytical methods and the interpretation of results.

Chromatographic separation of the analyses was performed with Hypersil GOLD aQ (20 × 2.1 mm, 12 μm) and Hypersil GOLD C18 (50 mm × 2.1 mm, 1.9μm) column from Thermo Scientific™ at a constant flow rate of 300 μL min^−1^. Online preconcentration using column switching was applied as a means to minimize sample pre-treatment and shorten analysis time. A Thermo Scientific™ EQuan MAX™ online sample concentration UHPLC-MS/MS system equipped with a Thermo Scientific™ Hypersil GOLD aQ™ preconcentration column (20 × 2.1 mm, 12 μm particle size) and a Thermo Scientific™ Hypersil GOLD™ analytical column (50 × 2.1 mm, 1.9 μm particle size) was used. The injection volume was 1 mL and the column temperature was maintained at 20 °C.

The mobile phase consisted of (A) ultra-pure water with 0.1% (*v*/*v*) formic acid and (B) acetonitrile with 0.1% (*v*/*v*) formic acid; gradient elution program operated as follows: the gradient started with 2% mobile phase B and 98% mobile phase A until time 1.2 min; in the range 1.2–1.6 min, mobile phase B increased from 2% to 100% and remained at 100% in the range 1.6–9.0 min; in the interval 9–11 min, mobile phase B decreased from 100% to 2% and mobile phase A increased from 0 to 98%. Between 11 and 14 min, mobile phase B remained at 2% and mobile phase A at 98%. All target compounds were eluted from the column within 14 min. The flow from the LC column was transferred to a triple quadrupole mass spectrometer equipped with an ESI source. Mass spectrometric analyses were performed in a triple quadrupole mass spectrometer equipped with an electrospray ionization (ESI) source that operated in the positive ionization mode. Ionization mode: Heated Electrospray (H-ESI); Scan type: SRM Polarity: Positive ion mode; Spray voltage [V]: 3400 positive Ion; sweep gas pressure [arb]: 0; Vaporizer temperature [°C]: 300; Sheath gas pressure [arb]: 30; Aux gas pressure [arb]: 10; Capillary temperature [°C]: 350; Collision gas pressure [mTorr]: 1.5; Cycle time [s]: 1; and Peak width: Q1/Q3 the full width of a peak at half its maximum height (FWHM) of 0.70 Da [38,39,40,41,42,43,44,45].

The optimized ionization mode, fragmentation voltages, collision energies and chromatographic retention times for each analyte are summarized in Table 4.

## 5. Conclusions

Veterinary antibiotics are a major source of environmental contamination. This study presents a complex investigation of exposure and environmental effects of a powdered veterinary medicinal product administered in the drinking water of intensively reared piglets, chickens and turkeys containing 250 mg/g penicillin G. Environmental Impact Assessment, carried out according to the EMEA guideline shows that the administration of this veterinary product does not pose any risk to the environment (R ˂ 1). Penicillin G concentrations detected in surface waters by SPE-Online-UHPLC-MS/MS were compared with the concentrations estimated according to the EMEA guideline. The results demonstrated that the estimated penicillin G concentration values (37.66 µg/L) are much higher than those determined in surface waters (0.032 µg/L).

## Figures and Tables

**Table 1:** Calculated predictable concentrations of penicillin G in soil, groundwater, surface water and sediment.

PEC		Penicillin G
soil		868.88 µg/kg
ground/	surface water	28.24/37.66 µg/kg
sediment		1721 µg/kg

**Table 2:** Estimated no-effect concentration (PNEC).

Compartment	Eco-Toxicology Dose Descriptors	AF	PNEC Value
water	NOEC:10 mg/L	10	1 mg/L
microorganism	EC50:100 mg/L	100	1 mg/L
soil	NOEC:2000 mg/kg	100	20 mg/kg

**Table 3:** Calculated risk, R, of penicillin G.

Compartment	PEC	PNEC	R (PEC/PNEC) Value
ground/surface water	0.028/0.037 mg/L	1 mg/L	0.028/0.037
soil	0.868 mg/kg	7.69 mg/kg	0.113
sediment	1.721 mg/kg	9.93 mg/kg	0.173

**Table 4:** Selected reaction monitoring transitions.

Compound	Retention Time (min)	Polarity	Precursor Ion (*m*/*z*)	Product Ion (*m*/*z*)	Collision Energy (V)
Penicillin G	5.94	Positive	335.2	176	14.1
				160	13
				114	31.5

## Data Availability

Not applicable.

## Sample Availability

Samples of the compounds are available from the authors.
